# Ethyl {4-[(1,5-dimethyl-2,4-dioxo-2,3,4,5-tetra­hydro-1*H*-1,5-benzo­diazepin-3-yl)meth­yl]-1,2,3-triazol-1-yl}acetate

**DOI:** 10.1107/S1600536810044120

**Published:** 2010-10-31

**Authors:** Rachida Dardouri, Youssef Kandri Rodi, Sonia Ladeira, El Mokhtar Essassi, Seik Weng Ng

**Affiliations:** a Laboratoire de Chimie Organique Hétérocyclique, Pôle de Compétences Pharmacochimie, Université Mohammed V-Agdal, BP 1014 Avenue Ibn Batout, Rabat, Morocco; b Service Commun Rayons-X, Laboratoire de Chimie de Coordination, 205 Route de Narbonne, Toulouse, France; c Department of Chemistry, University of Malaya, 50603 Kuala Lumpur, Malaysia

## Abstract

In the title compound, C_18_H_21_N_5_O_4_, the diazepine ring adopts a boat conformation with the triazolylmethyl-bearing C atom as the prow and the C atoms at the ring junction as the stern.

## Related literature

For the structure of 1,5-dimethyl-3-propargyl-1,5-benzodiazepine-2,4-dione, see: Dardouri *et al.* (2010 ▶).
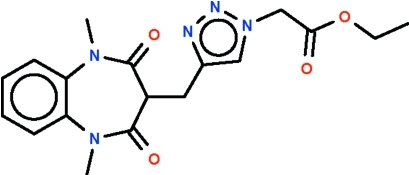


## Experimental

### 

#### Crystal data

C_18_H_21_N_5_O_4_
*M* *_r_* = 371.40
Monoclinic, 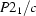

*a* = 8.5452 (2) Å
*b* = 15.9993 (5) Å
*c* = 13.9215 (4) Å
β = 106.853 (1)°
*V* = 1821.56 (9) Å^3^
*Z* = 4
Mo *K*α radiation
μ = 0.10 mm^−1^
*T* = 293 K
0.40 × 0.10 × 0.05 mm

#### Data collection

Bruker X8 APEXII diffractometer
15511 measured reflections
4129 independent reflections
2909 reflections with *I* > 2σ(*I*)
*R*_int_ = 0.037
Standard reflections: 0

#### Refinement

*R*[*F* ^2^ > 2σ(*F* ^2^)] = 0.045
*wR*(*F* ^2^) = 0.124
*S* = 1.03
4129 reflections
247 parameters
H-atom parameters constrained
Δρ_max_ = 0.30 e Å^−3^
Δρ_min_ = −0.31 e Å^−3^

### 

Data collection: *APEX2* (Bruker, 2008 ▶); cell refinement: *SAINT* (Bruker, 2008 ▶); data reduction: *SAINT*; program(s) used to solve structure: *SHELXS97* (Sheldrick, 2008 ▶); program(s) used to refine structure: *SHELXL97* (Sheldrick, 2008 ▶); molecular graphics: *X-SEED* (Barbour, 2001 ▶); software used to prepare material for publication: *publCIF* (Westrip, 2010 ▶).

## Supplementary Material

Crystal structure: contains datablocks global, I. DOI: 10.1107/S1600536810044120/bt5398sup1.cif

Structure factors: contains datablocks I. DOI: 10.1107/S1600536810044120/bt5398Isup2.hkl

Additional supplementary materials:  crystallographic information; 3D view; checkCIF report

## Figures and Tables

**Fig. 1.**
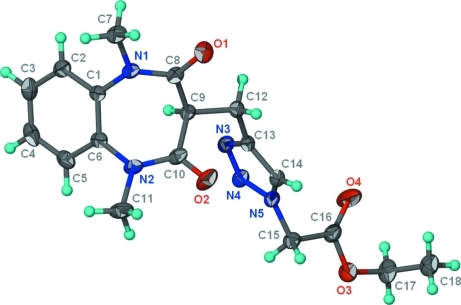
Anisotropic displacement ellipsoid plot (Barbour, 2001) of C18H21N5O4 at the 50% probability level; hydrogen atoms are drawn as arbitrary radius.

## supplementary crystallographic information

### Comment

1,5-Dimethyl-3-propargyl-1,5-benzodiazepine-2,4-dione, whose synthesis was reported recently (Dardouri *et al.*, 2010), possess an acetylenic linkage that can be exploited for the synthesis of other 1,5-benzodiazepine-2,4-dione derivatives. In this study, the compound is reacted with ethyl 2-azidoacetate to yield the title compound (Scheme I, Fig. 1). The ester provides three nitrogen atoms necessary for the formation of the triazolyl ring.

### Experimental

To a solution of 3-propargyl-1,5-dimethyl-1,5-benzodiazepine-2,4-dione (0.23 g,1 mmol) in a *t*-butyl alcohol/water mixture (1:2, 8 ml) was added copper sulfate pentahydrate (0.25 g,1 mmol), sodium ascorbate (0.29 g, 2 mmol) and ethyl 2-azidoacetate (0.64 g, 5 mmol). The mixture was stirred for two hours. Water (20 ml) was added and the organic compound was extracted with ethyl acetate (2 *x* 20 ml). The extracts were washed with brine and then dried over sodium sulfate. The compound was recrystallized from an *n*-hexane/ethyl acetate mixture to give colorless crystals.

### Refinement

H-atoms were placed in calculated positions (C—H 0.93–0.98 Å) and were included in the refinement in the riding model approximation, with *U*(H) set to 1.2–1.5*U*_eq_(C).

### Crystal data

**Table:**

C_18_H_21_N_5_O_4_	*F*(000) = 784
*M**_r_* = 371.40	*D*_x_ = 1.354 Mg m^−^^3^
Monoclinic, *P*2_1_/*c*	Mo *K*α radiation, λ = 0.71073 Å
Hall symbol: -P 2ybc	Cell parameters from 3798 reflections
*a* = 8.5452 (2) Å	θ = 2.5–26.4°
*b* = 15.9993 (5) Å	µ = 0.10 mm^−^^1^
*c* = 13.9215 (4) Å	*T* = 293 K
β = 106.853 (1)°	Prism, colorless
*V* = 1821.56 (9) Å^3^	0.40 × 0.10 × 0.05 mm
*Z* = 4	

### Data collection

**Table:**

Bruker X8 APEXII diffractometer	2909 reflections with *I* > 2σ(*I*)
Radiation source: fine-focus sealed tube	*R*_int_ = 0.037
graphite	θ_max_ = 27.5°, θ_min_ = 2.8°
φ and ω scans	*h* = −10→10
15511 measured reflections	*k* = −20→20
4129 independent reflections	*l* = −18→18

### Refinement

**Table:**

Refinement on *F*^2^	Primary atom site location: structure-invariant direct methods
Least-squares matrix: full	Secondary atom site location: difference Fourier map
*R*[*F*^2^ > 2σ(*F*^2^)] = 0.045	Hydrogen site location: inferred from neighbouring sites
*wR*(*F*^2^) = 0.124	H-atom parameters constrained
*S* = 1.03	*w* = 1/[σ^2^(*F*_o_^2^) + (0.052*P*)^2^ + 0.6008*P*] where *P* = (*F*_o_^2^ + 2*F*_c_^2^)/3
4129 reflections	(Δ/σ)_max_ = 0.001
247 parameters	Δρ_max_ = 0.30 e Å^−^^3^
0 restraints	Δρ_min_ = −0.31 e Å^−^^3^

### Fractional atomic coordinates and isotropic or equivalent isotropic displacement parameters (Å^2^)

**Table:**

	*x*	*y*	*z*	*U*_iso_*/*U*_eq_	
O1	0.56199 (17)	0.18555 (8)	0.67466 (9)	0.0438 (3)	
O2	0.81954 (18)	0.21921 (9)	0.49678 (10)	0.0505 (4)	
O3	0.41269 (16)	0.12018 (9)	−0.04809 (9)	0.0440 (3)	
O4	0.25337 (19)	0.10260 (14)	0.05096 (12)	0.0829 (7)	
N1	0.78013 (17)	0.10102 (8)	0.73772 (10)	0.0285 (3)	
N2	0.96549 (17)	0.11692 (10)	0.59493 (10)	0.0344 (4)	
N3	0.52214 (17)	0.02996 (9)	0.35097 (10)	0.0314 (3)	
N4	0.52862 (17)	0.02234 (9)	0.25819 (10)	0.0328 (3)	
N5	0.52498 (17)	0.10028 (9)	0.22117 (10)	0.0291 (3)	
C1	0.88147 (19)	0.03536 (10)	0.72159 (12)	0.0271 (4)	
C2	0.8961 (2)	−0.03761 (11)	0.77820 (13)	0.0347 (4)	
H2	0.8390	−0.0426	0.8255	0.042*	
C3	0.9939 (2)	−0.10250 (12)	0.76516 (15)	0.0421 (5)	
H3	1.0037	−0.1505	0.8042	0.050*	
C4	1.0772 (2)	−0.09629 (13)	0.69423 (15)	0.0449 (5)	
H4	1.1407	−0.1407	0.6840	0.054*	
C5	1.0662 (2)	−0.02442 (13)	0.63862 (13)	0.0395 (5)	
H5	1.1237	−0.0205	0.5914	0.047*	
C6	0.97042 (19)	0.04275 (11)	0.65161 (12)	0.0300 (4)	
C7	0.7764 (2)	0.11896 (12)	0.84068 (13)	0.0385 (4)	
H7A	0.7659	0.1781	0.8486	0.058*	
H7B	0.8758	0.0996	0.8875	0.058*	
H7C	0.6849	0.0909	0.8531	0.058*	
C8	0.6620 (2)	0.13566 (10)	0.66069 (12)	0.0301 (4)	
C9	0.6685 (2)	0.11352 (10)	0.55590 (11)	0.0263 (3)	
H9	0.6798	0.0528	0.5514	0.032*	
C10	0.8228 (2)	0.15520 (11)	0.54480 (12)	0.0332 (4)	
C11	1.1183 (2)	0.15061 (15)	0.58176 (15)	0.0499 (5)	
H11A	1.1209	0.2101	0.5909	0.075*	
H11B	1.1237	0.1378	0.5154	0.075*	
H11C	1.2100	0.1258	0.6304	0.075*	
C12	0.5152 (2)	0.14153 (11)	0.47517 (12)	0.0314 (4)	
H12A	0.4195	0.1194	0.4904	0.038*	
H12B	0.5083	0.2020	0.4755	0.038*	
C13	0.51605 (19)	0.11257 (10)	0.37318 (12)	0.0274 (3)	
C14	0.5173 (2)	0.15758 (11)	0.29023 (12)	0.0295 (4)	
H14	0.5136	0.2154	0.2830	0.035*	
C15	0.5444 (2)	0.11311 (12)	0.12218 (12)	0.0339 (4)	
H15A	0.6163	0.0703	0.1096	0.041*	
H15B	0.5963	0.1668	0.1207	0.041*	
C16	0.3849 (2)	0.11069 (11)	0.03967 (13)	0.0366 (4)	
C17	0.2699 (3)	0.11868 (14)	−0.13697 (14)	0.0487 (5)	
H17A	0.1960	0.0748	−0.1294	0.058*	
H17B	0.3051	0.1058	−0.1956	0.058*	
C18	0.1815 (2)	0.19999 (12)	−0.15270 (14)	0.0435 (5)	
H18A	0.0900	0.1969	−0.2121	0.065*	
H18B	0.2542	0.2436	−0.1603	0.065*	
H18C	0.1431	0.2120	−0.0958	0.065*	

### Atomic displacement parameters (Å^2^)

**Table:**

	*U*^11^	*U*^22^	*U*^33^	*U*^12^	*U*^13^	*U*^23^
O1	0.0564 (9)	0.0414 (7)	0.0342 (7)	0.0164 (6)	0.0141 (6)	−0.0056 (6)
O2	0.0584 (9)	0.0477 (8)	0.0410 (8)	−0.0153 (7)	0.0074 (7)	0.0153 (6)
O3	0.0389 (7)	0.0690 (9)	0.0205 (6)	0.0060 (6)	0.0030 (5)	−0.0024 (6)
O4	0.0371 (9)	0.1597 (19)	0.0459 (10)	−0.0232 (10)	0.0022 (7)	0.0413 (11)
N1	0.0337 (8)	0.0327 (7)	0.0201 (7)	−0.0024 (6)	0.0091 (6)	−0.0031 (5)
N2	0.0301 (8)	0.0491 (9)	0.0242 (7)	−0.0106 (7)	0.0080 (6)	0.0014 (6)
N3	0.0320 (8)	0.0344 (8)	0.0264 (7)	−0.0013 (6)	0.0063 (6)	0.0015 (6)
N4	0.0352 (8)	0.0332 (8)	0.0274 (7)	−0.0031 (6)	0.0050 (6)	0.0005 (6)
N5	0.0287 (7)	0.0341 (8)	0.0223 (7)	−0.0019 (6)	0.0040 (5)	0.0016 (6)
C1	0.0246 (8)	0.0334 (8)	0.0217 (8)	−0.0037 (7)	0.0043 (6)	−0.0038 (6)
C2	0.0307 (9)	0.0398 (10)	0.0312 (9)	−0.0051 (7)	0.0053 (7)	0.0024 (7)
C3	0.0365 (10)	0.0369 (10)	0.0446 (11)	0.0011 (8)	−0.0013 (8)	0.0032 (8)
C4	0.0312 (10)	0.0519 (12)	0.0437 (11)	0.0111 (8)	−0.0017 (8)	−0.0094 (9)
C5	0.0256 (9)	0.0631 (13)	0.0273 (9)	0.0038 (8)	0.0035 (7)	−0.0092 (9)
C6	0.0239 (8)	0.0429 (10)	0.0203 (8)	−0.0053 (7)	0.0018 (6)	−0.0034 (7)
C7	0.0472 (11)	0.0469 (11)	0.0227 (9)	−0.0019 (9)	0.0119 (8)	−0.0072 (8)
C8	0.0376 (10)	0.0269 (8)	0.0262 (8)	−0.0007 (7)	0.0098 (7)	−0.0017 (6)
C9	0.0310 (9)	0.0263 (8)	0.0214 (8)	0.0005 (7)	0.0069 (6)	−0.0008 (6)
C10	0.0395 (10)	0.0387 (9)	0.0204 (8)	−0.0080 (8)	0.0069 (7)	−0.0015 (7)
C11	0.0375 (11)	0.0799 (15)	0.0324 (10)	−0.0239 (10)	0.0104 (8)	0.0009 (10)
C12	0.0314 (9)	0.0361 (9)	0.0259 (8)	0.0072 (7)	0.0072 (7)	0.0018 (7)
C13	0.0229 (8)	0.0319 (8)	0.0255 (8)	0.0013 (6)	0.0042 (6)	0.0024 (7)
C14	0.0299 (9)	0.0296 (8)	0.0272 (8)	0.0010 (7)	0.0051 (7)	0.0016 (7)
C15	0.0329 (9)	0.0448 (10)	0.0225 (8)	−0.0031 (8)	0.0055 (7)	0.0001 (7)
C16	0.0348 (10)	0.0418 (10)	0.0293 (9)	−0.0072 (8)	0.0033 (7)	0.0055 (7)
C17	0.0509 (12)	0.0617 (13)	0.0231 (9)	0.0071 (10)	−0.0056 (8)	−0.0067 (9)
C18	0.0438 (11)	0.0441 (11)	0.0352 (10)	−0.0068 (9)	−0.0002 (8)	0.0007 (8)

### Geometric parameters (Å, °)

**Table:**

O1—C8	1.225 (2)	C5—H5	0.9300
O2—C10	1.219 (2)	C7—H7A	0.9600
O3—C16	1.319 (2)	C7—H7B	0.9600
O3—C17	1.464 (2)	C7—H7C	0.9600
O4—C16	1.186 (2)	C8—C9	1.518 (2)
N1—C8	1.360 (2)	C9—C12	1.525 (2)
N1—C1	1.420 (2)	C9—C10	1.524 (2)
N1—C7	1.471 (2)	C9—H9	0.9800
N2—C10	1.362 (2)	C11—H11A	0.9600
N2—C6	1.419 (2)	C11—H11B	0.9600
N2—C11	1.473 (2)	C11—H11C	0.9600
N3—N4	1.3145 (19)	C12—C13	1.495 (2)
N3—C13	1.362 (2)	C12—H12A	0.9700
N4—N5	1.3462 (19)	C12—H12B	0.9700
N5—C14	1.344 (2)	C13—C14	1.364 (2)
N5—C15	1.450 (2)	C14—H14	0.9300
C1—C2	1.394 (2)	C15—C16	1.507 (2)
C1—C6	1.404 (2)	C15—H15A	0.9700
C2—C3	1.377 (3)	C15—H15B	0.9700
C2—H2	0.9300	C17—C18	1.489 (3)
C3—C4	1.378 (3)	C17—H17A	0.9700
C3—H3	0.9300	C17—H17B	0.9700
C4—C5	1.374 (3)	C18—H18A	0.9600
C4—H4	0.9300	C18—H18B	0.9600
C5—C6	1.394 (3)	C18—H18C	0.9600
			
C16—O3—C17	116.81 (15)	C10—C9—H9	108.9
C8—N1—C1	121.62 (13)	O2—C10—N2	122.21 (16)
C8—N1—C7	117.89 (14)	O2—C10—C9	122.71 (16)
C1—N1—C7	119.01 (14)	N2—C10—C9	115.05 (15)
C10—N2—C6	122.59 (14)	N2—C11—H11A	109.5
C10—N2—C11	117.80 (16)	N2—C11—H11B	109.5
C6—N2—C11	119.40 (15)	H11A—C11—H11B	109.5
N4—N3—C13	109.17 (13)	N2—C11—H11C	109.5
N3—N4—N5	106.71 (13)	H11A—C11—H11C	109.5
C14—N5—N4	111.01 (13)	H11B—C11—H11C	109.5
C14—N5—C15	128.78 (14)	C13—C12—C9	111.66 (13)
N4—N5—C15	119.93 (14)	C13—C12—H12A	109.3
C2—C1—C6	119.01 (15)	C9—C12—H12A	109.3
C2—C1—N1	119.29 (15)	C13—C12—H12B	109.3
C6—C1—N1	121.68 (15)	C9—C12—H12B	109.3
C3—C2—C1	121.03 (17)	H12A—C12—H12B	107.9
C3—C2—H2	119.5	C14—C13—N3	108.06 (15)
C1—C2—H2	119.5	C14—C13—C12	130.07 (15)
C2—C3—C4	119.94 (18)	N3—C13—C12	121.82 (14)
C2—C3—H3	120.0	N5—C14—C13	105.05 (15)
C4—C3—H3	120.0	N5—C14—H14	127.5
C5—C4—C3	119.93 (18)	C13—C14—H14	127.5
C5—C4—H4	120.0	N5—C15—C16	113.16 (14)
C3—C4—H4	120.0	N5—C15—H15A	108.9
C4—C5—C6	121.26 (17)	C16—C15—H15A	108.9
C4—C5—H5	119.4	N5—C15—H15B	108.9
C6—C5—H5	119.4	C16—C15—H15B	108.9
C5—C6—C1	118.78 (16)	H15A—C15—H15B	107.8
C5—C6—N2	119.78 (15)	O4—C16—O3	124.56 (17)
C1—C6—N2	121.44 (15)	O4—C16—C15	125.75 (17)
N1—C7—H7A	109.5	O3—C16—C15	109.69 (15)
N1—C7—H7B	109.5	O3—C17—C18	111.90 (16)
H7A—C7—H7B	109.5	O3—C17—H17A	109.2
N1—C7—H7C	109.5	C18—C17—H17A	109.2
H7A—C7—H7C	109.5	O3—C17—H17B	109.2
H7B—C7—H7C	109.5	C18—C17—H17B	109.2
O1—C8—N1	122.20 (15)	H17A—C17—H17B	107.9
O1—C8—C9	121.75 (15)	C17—C18—H18A	109.5
N1—C8—C9	115.91 (14)	C17—C18—H18B	109.5
C8—C9—C12	112.12 (13)	H18A—C18—H18B	109.5
C8—C9—C10	105.62 (13)	C17—C18—H18C	109.5
C12—C9—C10	112.32 (14)	H18A—C18—H18C	109.5
C8—C9—H9	108.9	H18B—C18—H18C	109.5
C12—C9—H9	108.9		
			
C13—N3—N4—N5	−0.60 (17)	O1—C8—C9—C10	107.96 (18)
N3—N4—N5—C14	0.41 (17)	N1—C8—C9—C10	−67.92 (18)
N3—N4—N5—C15	174.79 (13)	C6—N2—C10—O2	179.53 (16)
C8—N1—C1—C2	−127.81 (17)	C11—N2—C10—O2	−5.9 (3)
C7—N1—C1—C2	38.0 (2)	C6—N2—C10—C9	1.3 (2)
C8—N1—C1—C6	53.7 (2)	C11—N2—C10—C9	175.88 (15)
C7—N1—C1—C6	−140.50 (16)	C8—C9—C10—O2	−103.15 (18)
C6—C1—C2—C3	−1.1 (2)	C12—C9—C10—O2	19.4 (2)
N1—C1—C2—C3	−179.65 (15)	C8—C9—C10—N2	75.09 (17)
C1—C2—C3—C4	−0.9 (3)	C12—C9—C10—N2	−162.40 (14)
C2—C3—C4—C5	1.8 (3)	C8—C9—C12—C13	−174.76 (14)
C3—C4—C5—C6	−0.7 (3)	C10—C9—C12—C13	66.48 (18)
C4—C5—C6—C1	−1.3 (2)	N4—N3—C13—C14	0.58 (18)
C4—C5—C6—N2	178.22 (15)	N4—N3—C13—C12	−177.28 (14)
C2—C1—C6—C5	2.2 (2)	C9—C12—C13—C14	−117.25 (19)
N1—C1—C6—C5	−179.29 (15)	C9—C12—C13—N3	60.1 (2)
C2—C1—C6—N2	−177.35 (14)	N4—N5—C14—C13	−0.06 (18)
N1—C1—C6—N2	1.2 (2)	C15—N5—C14—C13	−173.80 (15)
C10—N2—C6—C5	131.51 (17)	N3—C13—C14—N5	−0.31 (18)
C11—N2—C6—C5	−43.0 (2)	C12—C13—C14—N5	177.32 (16)
C10—N2—C6—C1	−49.0 (2)	C14—N5—C15—C16	−97.7 (2)
C11—N2—C6—C1	136.53 (17)	N4—N5—C15—C16	89.08 (19)
C1—N1—C8—O1	171.63 (16)	C17—O3—C16—O4	−1.3 (3)
C7—N1—C8—O1	5.6 (2)	C17—O3—C16—C15	179.53 (16)
C1—N1—C8—C9	−12.5 (2)	N5—C15—C16—O4	2.8 (3)
C7—N1—C8—C9	−178.49 (14)	N5—C15—C16—O3	−178.05 (15)
O1—C8—C9—C12	−14.7 (2)	C16—O3—C17—C18	79.5 (2)
N1—C8—C9—C12	169.44 (14)		
